# Five *Leptospira* spp. Antibody Point-of-Care Tests in Healthy Dogs Reveal Different Results After Revaccination Against Leptospirosis

**DOI:** 10.3390/microorganisms13112604

**Published:** 2025-11-15

**Authors:** Katharina Gesa Schmitt, Michèle Bergmann, Hans van der Linden, Ahmed A. Ahmed, Reinhard K. Straubinger, Yury Zablotski, Katrin Hartmann

**Affiliations:** 1 LMU Small Animal Clinic, Centre for Clinical Veterinary Medicine, Faculty of Veterinary Medicine, LMU Munich, Veterinaerstrasse 13, 80539 Munich, Germany; k.schmitt@medizinische-kleintierklinik.de (K.G.S.); y.zablotski@med.vetmed.uni-muenchen.de (Y.Z.); hartmann@lmu.de (K.H.); 2 Expertise Centre for Reference and Research on Leptospirosis, WOAH Reference Laboratory for Leptospirosis, University Medical Center, Meibergdreef 39, 1105 AZ Amsterdam, The Netherlands; h.vanderlinden@amsterdamumc.nl (H.v.d.L.); a.ahmed@amsterdamumc.nl (A.A.A.); 3 Institute of Bacteriology and Mycology, Department of Veterinary Sciences, Faculty of Veterinary Medicine, LMU Munich, Sonnenstrasse 24, 85764 Oberschleissheim, Germany; r.straubinger@lmu.de

**Keywords:** canine, vaccination, IgM, IgG, MAT, WITNESS^®^ Lepto, FASTest^®^ LEPTOSPIRA IgM, Test-it^TM^ Leptospirosis, ImmunoComb^®^ Canine *Leptospira* Test Kit, SNAP^®^ Lepto

## Abstract

The microscopic agglutination test (MAT) is the diagnostic standard for canine leptospirosis. However, it is a time-consuming process and does not differentiate between infection- and vaccine-induced antibodies. Canine *Leptospira* spp.-specific antibody point-of-care (POC) tests provide the rapid detection of immunoglobulin M (IgM) and/or G (IgG). IgM POC tests are considered to become negative more rapidly after vaccination, making them more effective at diagnosing leptospirosis in not-recently vaccinated dogs. This study analysed 582 serum samples of 97 healthy dogs using five different POC tests and the MAT before vaccination and 2, 4, 12, 26, and 52 weeks afterwards. Among the POC tests, three detected IgM antibodies, one detected IgG antibodies, and one detected both IgM and IgG. The results were analysed using mixed-effects logistic regression. Before vaccination, only 2/291 IgM tests were positive (0.7%), compared to 45/194 IgG tests (23.2%). All the POC tests became positive after vaccination, but IgM positivity occurred significantly less frequently (59/1746), especially >4 weeks post-vaccination (7/59 positive results), with 94.5–99.6% specificity compared to 41.4–77.8% in IgG tests. These findings support the use of IgM POC tests in vaccinated dogs, while IgG POC tests are more difficult to interpret.

## 1. Introduction

Leptospirosis is a zoonotic infectious disease of global importance [1]. Due to its increasing incidence among different animal species, including dogs, it is considered a re-emerging disease in many areas worldwide [2,3,4,5]. The pathogens that cause leptospirosis are spirochetes belonging to the genus *Leptospira* [6]. Pathogenic *Leptospira* spp. colonize the renal tubules of infected hosts, leading to environmental contamination via urinary shedding [7]. Both humans and animals can become infected when mucous membranes or skin lesions come into contact with leptospires [1,8]. Infected dogs can first show unspecific signs, such as lethargy, anorexia, and fever. However, the disease can rapidly progress to acute kidney injury (AKI) and other organ failures [7,8], which is one of the reasons why vaccination against leptospirosis is considered a canine core component [8,9]. Studies have shown that vaccination in dogs can lead to a long-term high-level antibody response, as measured by the microscopic agglutination test (MAT)—not only against vaccine serogroups [10], but also against serovars from non-vaccine serogroups [10,11,12,13]. The elevated levels of antibody titres observed in response to serovars not included in the vaccine can be explained on immunological grounds. It has been demonstrated that certain *Leptospira* spp. serovars share antigenic determinants due to their lipopolysaccharide (LPS) antigens [14]. Thus, vaccine antigens can also stimulate B-cell clonal lines that recognise structurally related epitopes. This, however, poses a problem, since antibodies induced by the vaccine can complicate the diagnosis.

There are several possibilities for confirming a clinical diagnosis of leptospirosis, e.g., the MAT, point-of-care (POC) tests, polymerase chain reaction (PCR), or darkfield microscopy. All methods have limitations [8,15]. Antibody detection is important, as the PCR results are often negative, particularly in dogs pre-treated with antibiotics [16]. The MAT detects antibodies against leptospiral serogroups and has been considered the reference method for diagnosing leptospirosis [8,15,17]. However, the MAT cannot differentiate between antibodies resulting from an infection and those induced by vaccination [13,18,19]. Additionally, the test results can be negative if the testing is performed as early as two to 14 days after infection [8].

An alternative diagnostic approach is the use of the recently developed POC tests, which can be used in-house for antibody detection against *Leptospira* spp. [20,21,22,23,24]. The testing principle is either an enzyme-linked immunosorbent assay (ELISA) or an immunochromatographic process. One major advantage of POC tests is the feature that the results are obtained within minutes, a fact that is highly relevant due to the zoonotic risk for dog owners and veterinary staff [17,25], as well as the potential need for intensive medical treatment [8,15,26]. In addition, there are some POC tests that detect only the IgM isotype. Their advantage is that IgM antibodies are generally only detectable for a shorter period after contact, thus indicating an acute infection or recent vaccination, in contrast to IgG antibodies that remain positive for months following an infection or vaccination [11]. So far, there have been studies on three of these POC tests (WITNESS^®^ Lepto, Zoetis; SNAP^®^ Lepto, IDEXX; and Test-it^TM^ Leptospirosis, Life Assay) [20,21,22,23,24], but no studies have investigated the performance of all available POC tests. Therefore, the aim of the present study was to compare the results of all five commercially available *Leptospira* spp.-specific antibody POC tests in Germany (WITNESS^®^ Lepto, Zoetis; FASTest^®^ LEPTOSPIRA IgM, MegaCor; Test-it^TM^ Leptospirosis, Life Assay; ImmunoComb^®^ Canine *Leptospira* Test Kit, Biogal; and SNAP^®^ Lepto, IDEXX) with the MAT results after vaccination using sera from 97 healthy, revaccinated dogs collected over 52 weeks. The purpose of this study was to specifically analyse whether and to what extent the results of POC tests are influenced by vaccine-induced antibodies in a field setting that includes dogs who received a primary vaccination in the past and have since been revaccinated, because this mimics the situation for most adult dogs in veterinary practices. The hypothesis was that POC tests, especially those detecting only IgM antibodies, would turn negative much faster than the MAT after vaccination, and thus, would be more useful for diagnosing leptospirosis infections in dogs that have not recently received a vaccine.

## 2. Materials and Methods

### 2.1. Data Collection

This prospective study was approved by the government of Upper Bavaria (ethical approval code: ROB-55.2-2532.Vet_03-18-33). Healthy adult dogs (*n* = 97) that were presented to the LMU Small Animal Clinic’s preventive health care service for routine leptospirosis vaccination were enrolled. A 4-serovar vaccine (Nobivac^®^ L4, MSD Animal Health, Boxmeer, The Netherlands, Table 1) was used for vaccination.

The dogs were presented to the same clinician at week 0 (date of annual revaccination) and after 2, 4, 12, 26, and 52 weeks (allowing a ±2-day deviation) post-revaccination for a physical examination. At each time point, a total of 3 mL of serum was collected for antibody detection by the MAT (with a 12-serovar panel in six dilution levels) and five POC tests; the samples were frozen at −80 °C. The first sample (week 0) was taken before annual revaccination against leptospirosis. Vaccine-associated adverse events (VAAEs) such as lethargy, anorexia, or signs of pain were monitored at each time point. In addition, the owners were contacted up to 14 days after vaccination and asked about possible VAAEs.

### 2.2. Study Population

Only healthy dogs weighing at least 5 kg (range: 5–50 kg, average weight: 19.7 kg) were included. Their health status was ensured by their history and a physical examination. All the dogs had already received a primary vaccination with a 4-serovar (L4) vaccine (covering the serovars of 4 different serogroups) and had not received any immunosuppressive treatment within the previous 4 weeks.

The dogs’ age was between two and 14 years; 41.2% of the dogs were male (*n* = 40) and 58.8% were female (*n* = 57). The majority of the dogs lived in the city (*n* = 74 or 76.3%). About half of the dogs shared their home with at least one other dog (*n* = 52 or 53.6%), and 63.9% of the dogs (*n* = 62) had a travel history. Dogs that became ill or required immunosuppressive therapy during the study were excluded. This study population was already part of another study on canine leptospirosis [13].

### 2.3. MAT

The samples were analysed by the MAT, which was carried out at the WOAH Reference Laboratory for Leptospirosis of Amsterdam’s University Medical Center. The tests were conducted in accordance with applicable standards [16]. Briefly, 50 µL of phosphate-buffered saline (PBS) and 10 µL of sera were added to a well of a 96-well flat-bottomed plate.

A serial dilution was made across six wells, and 25 µL of a liquid culture with live *Leptospira* spp. organisms were added to each well, creating a final serum dilution series of 10 to 640. Both negative and positive controls were included. Every sample was tested for antibodies against the 4 vaccine serovars as well as against eight non-vaccine serovars (Table 2). The plates were incubated for two h at 30 °C and the results were obtained using a darkfield microscope. The highest dilution of serum at which at least 50.0% of the leptospires still showed agglutination was taken as the antibody titre for that sample and serovar [16]. The analyses were performed by the same person.

An MAT antibody titre of ≥10 (baseline titre) was considered positive in order to verify the existence of antibodies after revaccination. Additionally, a cut-off titre ≥ 80 (reference titre) was regarded as a positive MAT result according to the standards of the World Organisation of Animal Health (WOAH) for epidemiological studies [27] and was used for further analyses within this study.

### 2.4. POC Tests

The sera were analysed by the same person using five in-house POC tests commercially available in Germany, i.e., WITNESS^®^ Lepto, Zoetis (Lyon, France); FASTest^®^ LEPTOSPIRA IgM, MegaCor (Hörbranz, Austria)*;* Test-it^TM^ Leptospirosis, Life Assay (Cape Town, South Africa); ImmunoComb^®^ Canine *Leptospira* Test Kit, Biogal (Kibbutz Galed, Israel); SNAP^®^ Lepto, IDEXX (Hoofddorp, The Netherlands) (Table 3). POC tests of the same batch were used for each manufacturer. The tests were conducted according to the manufacturer’s operating instructions (Table 3).

### 2.5. Statistical Analysis

The statistical program R, version 4.4.0, was used for all the statistical analyses. Mixed-effects logistic regression analyses with the interaction between the tests and time points were performed to compare the antibody detection rates of the different POC tests and the MAT. Individual dogs were used as a random intercept to account for repeated measurements within the dogs. Comparative analyses were carried out via the “emmeans” R package, version 1.11.1, between individual tests within different time points (weeks 0, 2, 4, 12, 26, and 52), and between different time points within the POC tests. A *p*-value <0.05 was considered significant, but a Tukey correction for multiple comparisons within mixed-effects models was applied. The entire MAT panel analysed was used to calculate the specificity and sensitivity with both cut-off titres (baseline titre ≥ 10 and reference titre ≥ 80). The sensitivity, the specificity, and other confusion matrix metrics were determined compared to the gold standard MAT.

## 3. Results

At the beginning, 99 dogs entered the study. Of these, two were excluded: one due to a diagnosis of cancer and one due to systemic therapy with glucocorticoids. All the data and samples obtained from these two dogs up to that date were subsequently excluded from further analyses. This resulted in a final total of 97 dogs and 582 serum samples, as described in the Materials and Methods section. The samples were assigned individual numbers so that the time point at which each sample was drawn could be identified during the evaluation, but not the individual dog.

### 3.1. MAT Results

The antibodies prior to revaccination and the subsequent antibody response were measured by the MAT. Antibodies against both vaccine and non-vaccine serovars could be detected (Table 4a,b).

Of 97 dogs, 63 (64.9%) had detectable antibody titres against ≥1 vaccine serovars at week 0, ranging from 10 to 80. Furthermore, 23/97 dogs (23.7%) had antibodies (ranging from 10 to 40) against ≥1 non-vaccine serovars prior to revaccination.

After revaccination, in all dogs (97/97, 100.0%), antibodies against at least one vaccine serovar were detected. Maximum titres of 320 after revaccination were obtained. In 75/97 dogs (77.3%), antibodies against at least one non-vaccine serovar were detectable after revaccination, with titres ranging from 10 to 80. These included antibodies against the serovars Autumnalis, Pomona, Pyrogenes, Javanica, Saxkoebing, and Tarassovi.

The antibody titres were the highest 4 weeks after revaccination, with 73.2% (71/97 dogs) having detectable antibody titres against at least one vaccine serovar at all study time points until week 52. In 21/97 dogs (21.6%), MAT antibodies were only detectable until week 12 and were negative afterwards in all serovars.

### 3.2. POC Test Results

Both the practicability and the diagnostic outcomes, including the sensitivity and specificity, were assessed.

#### 3.2.1. Practicability

The tests WITNESS^®^ Lepto (Zoetis), FASTest^®^ LEPTOSPIRA IgM (MegaCor), and Test-it^TM^ Leptospirosis (Life Assay) were easy to perform, with only two processing steps each. The SNAP^®^ Lepto (IDEXX) required five simple processing steps that were also easy to perform. The results of these four tests were easy to interpret. There were clear control and test lines or spots in each of these tests. Only the ImmunoComb^®^ Canine *Leptospira* Test Kit (Biogal) was less user-friendly, as it required 14 processing steps and the results were sometimes difficult to interpret; in 8/582 cases (1.4%), the test results could not be interpreted because the test-spot colour intensity could not clearly be assigned to the provided scale. These tests, therefore, had to be repeated and then provided clear results so that they could be included into subsequent analyses. Due to the more complex implementations of the SNAP^®^ Lepto (IDEXX) and the ImmunoComb^®^ Canine *Leptospira* Test Kit (Biogal), both tests required more time until the test results could be read out. In addition, these tests had to be adjusted to room temperature before use, which required additional time (Table 5).

#### 3.2.2. Test Performance

The number of positive dogs per time point varied among the different POC tests (Table 6).

Two weeks after revaccination, a higher number of dogs were positive. The IgM-based POC tests yielded the majority of the positive results up to four weeks after revaccination (32 positives in week 2 and 18 positives in week 4), while only three or fewer positive results per time point were observed from week 12 onwards (<2.0% of samples tested positive).

Throughout the entire study period, significantly more dogs were positive at each time point with the ImmunoComb^®^ Canine *Leptospira* Test Kit (Biogal) and SNAP^®^ Lepto (IDEXX) (*p* ≤ 0.011). A significant difference was also observed between these tests (*p* < 0.005). Furthermore, significant differences were found when comparing all five POC tests, either across the full study period or at specific time points (Table 7). However, no significant differences were observed between the IgM-detecting POC tests at any time point (*p*-values ≥ 0.205), apart from Test-it^TM^ Leptospirosis (Life Assay) in week 2 in comparison to WITNESS^®^ (*p* = 0.011) and to FASTest^®^ (*p =* 0.045) (Table 7).

The determination of the POC tests’ sensitivity and specificity was based on a reference cut-off titre ≥ 80. Table 8 provides an overview of the sensitivity and specificity for each POC test. The proportion of positive samples for each POC test (prevalence) is included.

## 4. Discussion

Leptospirosis can be diagnosed using several methods, with the MAT still being considered the diagnostic gold standard. Studies have shown a high sensitivity and specificity for the MAT when testing paired sera [7,18,28], but it takes up to 14 days after infection to become positive, and thus, it is not adequate for making an early diagnosis [8]. Additionally, the MAT cannot differentiate between IgM and IgG antibodies, and the antibodies produced after vaccination cannot be distinguished from those after infection [10,13]. In this prospective study, serum samples from 97 healthy, adult dogs were collected at six defined time points over 52 weeks after revaccination with an L4 vaccine. The POC tests that detect only IgM antibodies were indeed mainly positive in weeks 2 (11.0%) and 4 (6.2%) after revaccination, whereas other POC tests that also or only detect IgG antibodies showed significantly more positive results throughout the whole study period (between 23.2% and 63.9% per time point). Even at week 0, a marked discrepancy was observed between the tests that detect IgM antibodies (WITNESS^®^ Lepto (Zoetis) and FASTest^®^ LEPTOSPIRA IgM (MegaCor) with 1/97 positive results each, and Test-it^TM^ Leptospirosis (Life Assay) all negative) and those POC tests that detect IgG antibodies, either exclusively (ImmunoComb^®^ Canine *Leptospira* Test Kit (Biogal) with 32/97 positive) or in combination with IgM (SNAP^®^ Lepto (IDEXX) with 13/97 positive). In the present study, 32 dogs tested positive in at least one of the IgM POC tests two weeks after vaccination, 18 dogs four weeks after vaccination, two dogs 12 weeks after vaccination, three dogs 26 weeks after vaccination, and two dogs 52weeks after vaccination. In another field study, IgM antibodies could also be detected by ELISA for a maximum of 10 weeks after vaccination [29]. The decline in IgM titres likely reflects the transient nature of the early humoral response, and the results support the hypothesis that IgM-detecting POC tests become negative relatively shortly after vaccination, although potential assay-dependent sensitivity differences in IgM detection have to be considered. Nevertheless, these tests are more suitable for detecting infection-associated antibodies than the MAT or IgG-detecting POC tests, at least in dogs that have not been recently vaccinated. As most adult dogs in veterinary practices are regularly vaccinated against canine leptospirosis, this study aimed to specifically analyse if POC tests are influenced by vaccine-induced antibodies in a field setting. The tests’ outcomes in samples of naïve dogs would also be very interesting and should be considered in future studies.

The present study showed one positive result for the WITNESS^®^ Lepto (Zoetis) and for the FASTest^®^ LEPTOSPIRA IgM (MegaCor) before revaccination, with each detecting IgM antibodies. Contrarily, Lizer and colleagues (2017), who tested 25 healthy dogs before vaccination with WITNESS^®^ Lepto (Zoetis), received only negative results [22]. Kodjo and colleagues (2016) tested the serum of 50 healthy dogs from a bank of clinical samples [21]. Among those, 38 dogs had been vaccinated within 12 months prior to the study, and still, all the results with WITNESS^®^ Lepto (Zoetis) were negative. The dog in the present study had no clinical signs of an acute infection with *Leptospira* spp. and had received its last vaccination 12 months earlier. In addition, the MAT results were negative for nine serovars, and three serovars had titres of 10 (lowest titre level). Thus, a false-positive test result in the WITNESS^®^ Lepto (Zoetis) seems most likely in the present study. The FASTest^®^ LEPTOSPIRA IgM (MegaCor) has not been utilized in studies before. In the present study, the dog with a positive FASTest^®^ LEPTOSPIRA IgM (MegaCor) test result in week 0 was negative for the eight serovars tested, had titres of 10 in three serovars, and had a titre of 40 against the serovar Javanica, measured by the MAT. This dog maintained MAT titres of 20 or 40 against the serovar Javanica throughout the study period, despite showing no clinical symptoms. Nevertheless, this titre could have resulted in a positive POC test. As this dog was the only one to show relatively high titres against the serovar Javanica and a subclinical infection seemed possible, a PCR test was performed on urine obtained by cystocentesis after the study had finished. This was negative. Thus, a false-positive test result seems most likely.

The IgG-detecting ImmunoComb^®^ Canine *Leptospira* Test Kit (Biogal) (32 positive results) and IgM/IgG-detecting SNAP^®^ Lepto (IDEXX) (13 positive results) were significantly more likely to be positive before revaccination, compared to the IgM-detecting POC tests. IgG antibodies could be caused by persistent vaccine antibodies or field infections (acute, subclinical, or previous infection), and are known to stay positive at least up to 365 days after vaccination or an infection [11,29]. Novak and colleagues (2023) examined the cellular and humoral immune responses in 15 dogs that had been vaccinated annually with an L4 vaccine [30]. A significant rise in the IgG antibody titres was evident following each booster vaccination, while the IgM antibody titres exhibited a significant increase exclusively during the first year. This was interpreted as a class switch to IgG antibodies and as an indication of B-cell memory. Despite the absence of direct detection and the paucity of study data on B-cell memory in dogs, and also humans, following leptospirosis vaccination, studies on B-cell memory formation after other bacterial and viral vaccinations in humans have demonstrated that memory B cells play a significant role in long-term immunity [31,32], resulting in long-term IgG antibodies. False-positive results are also possible. Seven dogs tested positive throughout the study period using both the SNAP^®^ Lepto (IDEXX) and ImmunoComb^®^ Canine *Leptospira* Test Kit (Biogal). In most of these dogs, MAT antibodies against vaccine serovars with titres between 10 and 40 were detected, which were most likely derived from previous vaccinations. Thus, the IgG antibodies detected by these two POC tests likely represented vaccine-derived antibodies. One of these seven dogs, however, had no antibodies detected by the MAT at week 0, which is why the POC test results should be considered false positives. It is also possible that the dog had antibodies against a serovar that was not included in the MAT panel. One of the dogs had a high antibody titre of 160 against the serovar Canicola. Dogs are considered reservoir hosts for the serovar Canicola [33]. Studies in vaccinated and unvaccinated healthy dogs have detected antibodies against the serovar Canicola by the MAT, with measurable titres between 100 and 6400 [34,35]. Dogs that had been vaccinated within the last six months showed high antibody titres between 100 and 400 against the serovar Canicola, which the authors of that study considered to be a possible result of previous vaccinations [35]. The dog with the high Canicola antibody titre in the present study was a six-year-old dog that had received primary immunisation as a puppy and had been vaccinated annually with an L4 vaccine since then. Persistent vaccine-induced antibodies are therefore also plausible in this case.

As expected, there were more positive dogs two weeks after revaccination in all the POC tests, which is in accordance with the results of Sant’Anna and colleagues (2022), who recorded an increase in antibodies 15 days after vaccination, as measured by the MAT and IgG ELISA [11]. However, it is surprising that, in the present study, only 32 out of 97 dogs were positive after two weeks, and 18 dogs were positive after four weeks in the IgM POC tests WITNESS^®^ Lepto (Zoetis) (*n* = 18 and *n* = 10), FASTest^®^ LEPTOSPIRA IgM (MegaCor) (*n* = 13 and *n* = 7), and Test-it^TM^ Leptospirosis (Life Assay) (*n* = 1 and *n* = 1), respectively. However, IgM antibodies are an expected reaction to vaccination. Indeed, the antibodies measured by the MAT suggested that all 97 dogs responded to vaccination (as measured by the baseline titre). All the dogs that tested negative in the IgM POC tests showed an increase in antibody titres, as measured by the MAT from week 0 (before revaccination) to week 2 of at least one dilution step, but many dogs only had low titres. In the MAT, only 11 dogs had high antibody titres ≥ 80 in week 2, and 6 dogs also had high titres in week 4. Thus, the antibody titres of many dogs might have been too low to be detected by the IgM POC tests. It is likely that the vaccine used in the present study did not induce a strong antibody response. A primarily cellular immune response cannot be excluded, as indicated by a recently published in vitro study [30].

Lizer and colleagues (2017) observed more positive results with WITNESS^®^ Lepto (Zoetis) in week 4 in the group of vaccinated dogs (16/25 positive) [22]. However, in contrast to the present study, these dogs were vaccinated twice within the four weeks prior to testing. This could explain the higher percentage of positive dogs in week 4 (64.0% positive sera compared to 18.6% in the present study) and week 12 (24.0% positive sera compared to 1.0% in the present study).

With the FASTest^®^ LEPTOSPIRA IgM (MegaCor), 13 dogs tested positive in week 2 and another seven dogs tested positive in weeks 2 and 4 after vaccination. Thus, this test led to fewer positive results than WITNESS^®^ Lepto (Zoetis). Surprisingly, Test-it^TM^ Leptospirosis (Life Assay) hardly showed any positive results during the study course. Two dogs were positive with the test in week 2 or 4, with corresponding MAT titres of 10 and 40, respectively. Other dogs had higher antibody titres, as measured by the MAT (80 against Icterohaemorrhagiae), and were not recognised as positive by the Test-it^TM^ Leptospirosis (Life Assay). According to the manufacturer’s instructions, the Test-it^TM^ Leptospirosis (Life Assay) contains the serovar Icterohaemorrhagiae, while the other POC tests contain additional serovars. Despite the considerable cross-reactivity exhibited by both IgM and IgG tests, this may well constitute the primary factor contributing to the observed discrepancy in the number of positive Test-it^TM^ Leptospirosis (Life Assay) results. Why the antibodies in the two positive dogs in this study were detected and others were not is unknown. However, the test cannot be recommended in its current form, as false-negative results are likely when compared to the MAT results, and there was a remarkable difference in the results compared to the other IgM POC tests.

The agglutination on which the MAT is based is triggered by both IgM and IgG *Leptospira* spp.-specific antibodies [5]. In the present study, there were very few positive dogs with IgM POC tests in weeks 12, 26, and 52 (only 0, 1, or 2 positive results per test and time point), but the IgG POC tests still showed many positive results (ImmunoComb^®^ Canine *Leptospira* Test Kit (Biogal): 66 positive results in week 12 and 42 in week 52; SNAP^®^ Lepto (IDEXX): 21 positive results in week 12 and 12 in week 52). To avoid the detection of vaccine-induced antibodies, IgM-detecting POC tests are therefore superior to POC tests that also detect IgG antibodies.

Antibodies were detectable by the MAT throughout the whole study course in most dogs (*n* = 52). Of the remaining 45 dogs, 22 dogs had detectable antibodies until week 26. An old field study from 1984 also demonstrated long-lasting IgG antibodies (up to 365 days) after regular vaccinations [29]. Most dogs also tested positive with the ImmunoComb^®^ Canine *Leptospira* Test Kit (Biogal) and SNAP^®^ Lepto (IDEXX) (Table 6), particularly in weeks 2, 4, and 12, when the highest antibody titres were detected by the MAT as well. Thus, positive results in these POC tests correlated well with those of the MAT, and they share the same disadvantages as the MAT.

Among 97 dogs, 12 individuals tested negative throughout the study period in the IgG-detecting POC test ImmunoComb^®^ Canine *Leptospira* Test Kit (Biogal). These were mainly young dogs with low MAT antibody titres. Therefore, this POC test had a similar disadvantage as the MAT. Of these dogs, 9/12 were also negative for SNAP^®^ Lepto (IDEXX).

The reference titre of 80 (Table 8) was applied in accordance to the WOAH standards for epidemiological studies (cut-off value between 50 and 100) [27]. In the present study, most dogs had low antibody titres, as measured by the MAT. Using the WOAH standard, the sensitivity of the POC tests ranged between 0.0% and 94.7% and the specificity ranged between 41.4% and 99.6%. The specificity was the decisive parameter for the question addressed in this study, as it reflects the extent to which the tests become positive due to vaccine-induced antibodies.

The specificity of the IgM-detecting tests ranged between 94.5% and 99.6%, while those of the ImmunoComb^®^ Canine *Leptospira* Test Kit (Biogal) (41.4%) and SNAP^®^ Lepto (IDEXX) (77.8%) were lower. Evaluations of WITNESS^®^ Lepto (Zoetis), Test-it^TM^ Leptospirosis (Life Assay), and SNAP^®^ Lepto (IDEXX) have been conducted in previous studies [20,21,22,23,24]. However, these studies did not concentrate on vaccinated dogs. Only Lizer and colleagues (2017) examined a group of dogs that had received primary immunisation, but without determining the sensitivity or specificity in these dogs [22]. Gloor and colleagues (2017) included 21/161 dogs that had been vaccinated with an L4 vaccine (containing serovars of the serogroups Canicola, Icterohaemorrhagiae, Grippotyphosa, and Australis) and evaluated the WITNESS^®^ Lepto (Zoetis) and Test-it^TM^ Leptospirosis (Life Assay) in comparison with the MAT [20]. The WITNESS^®^ Lepto (Zoetis) and Test-it^TM^ Leptospirosis (Life Assay) had a specificity of 100.0% (with a negative predictive value (NPV) of 74.0%) and 91.0% (with an NPV of 73.0%). The results of the present study showed similar specificities (94.5% with an NPV of 97.0% and 99.6% with an NPV of 96.7%). According to the manufacturer of Test-it^TM^ Leptospirosis (Life Assay), the test provides a specificity of 95.3%, resembling the results of the present study. There are no manufacturer specifications or comparable studies on the accuracy of the remaining POC tests published to date.

Parallel testing using the MAT as the reference method allowed the antibody titre of each dog to be determined and the POC tests to be evaluated on this basis. In terms of specificity, the present study on healthy dogs did not show any major differences when compared to a previous study that also included healthy, vaccinated dogs [20]. When comparing the specificities of the POC tests examined in the present study, the IgM-detecting POC tests had a higher specificity than the IgG-detecting POC tests. Taking the time progression into account, the IgM POC tests were more reliable in identifying healthy, vaccinated dogs as non-infected if the vaccination was administered longer than four weeks ago. Several previous clinical studies on infected dogs have already shown that the POC test WITNESS^®^ Lepto (Zoetis) is effective at detecting the immune response during the acute phase of a leptospirosis infection [20,21,22,23,24]. Therefore, a positive IgM POC test result in a dog that has not recently been vaccinated can be helpful in diagnosing leptospirosis, even in a regularly vaccinated dog. It is important to note that the persistence of IgM antibodies can extend to several weeks; therefore, these tests might not always be reliable in a recently vaccinated dog.

A regionally varying occurrence of different *Leptospira* spp. serovars might also contribute to the slightly varying specificities of these tests [8]. The participating dogs of the present study were mainly recruited in Munich, Germany, and the surrounding area, with the predominating leptospiral serovars Grippotyphosa (serogroup Grippotyphosa) and Saxkoebing (serogroup Sejroe) [36]. Antibodies against the serogroup Grippotyphosa (vaccine serovar) were detected in 94/97 dogs, especially in weeks 2 and 4, whereas antibodies against the serogroup Sejroe (non-vaccine serovar) were only detected in 1/97 dogs by the MAT.

Nevertheless, regional differences in the distribution of different serovars might have influenced the specificity data. In the present study, mainly antibodies against the vaccine serovars and also against the non-vaccine serovar Autumnalis were detected by the MAT. A study by Barr and colleagues (2005) demonstrated that vaccination with an L2 vaccine against the serovars Grippotyphosa and Pomona also led to the development of antibodies against the serovar Autumnalis [37]. Since the serovar Dadas of the serogroup Grippotyphosa is used in the vaccine, it does belong to the same serogroup and a cross-reaction is likely.

### Limitations

A gold standard measurement of IgM and IgG antibodies for a direct comparison with the POC test results was not performed. Additionally, the POC tests were carried out after vaccination with Nobivac^®^ L4 only, and not after other vaccines. The results could vary in dogs that are vaccinated with a different vaccine.

## 5. Conclusions

The present study confirmed the hypothesis that IgM-based POC tests become negative more rapidly than the MAT or IgG-based POC tests. The IgG-detecting POC tests were frequently positive, even up to 52 weeks after vaccination, most likely due to persisting vaccine antibodies. Thus, IgM-detecting POC tests are more suitable and can help provide a rapid diagnosis of leptospirosis in regularly revaccinated dogs, especially if the last vaccination was longer than four weeks ago. Given the low number of positive test results for the Test-it^TM^ Leptospirosis (Life Assay) compared to other IgM-detecting POC tests, the further evaluation of this test is indicated, as only healthy dogs were evaluated in the present study. In view of the fact that a false-negative result in a dog with leptospirosis can have severe consequences, further studies on the sensitivity of the IgM POC tests FASTest^®^ LEPTOSPIRA IgM (MegaCor) and Test-it^TM^ Leptospirosis (Life Assay) in confirmed cases of canine leptospirosis are recommended.

## Figures and Tables

**Table 1:** Active ingredients of the leptospirosis vaccine (Nobivac^®^ L4, MSD Animal Health, Boxmeer, The Netherlands) according to the manufacturer.

Inactivated *Leptospira* spp.	Serogroup	Serovar (Strain)	Amount of AG ^1^ in ELISA ^2^ Units
*L.* ^3^ *interrogans*	Canicola	Portland-vere (Ca-12-000)	3550–7100
	Icterohaemorrhagiae	Copenhageni (Ic-02-001)	290–1000
	Australis	Bratislava (As-05-073)	500–1700
*L. kirschneri*	Grippotyphosa	Dadas (Gr-01-005)	650–1300

^1^ AG, antigen; ^2^ ELISA, enzyme-linked immunosorbent assay; ^3^ *L*., *Leptospira.*

**Table 2:** Panel of strains and their serogroups used for microscopic agglutination test.

Serogroup	Serovar	Reference Strain
**Australis**	**Australis**	**Ballico**
Autumnalis	Autumnalis	Akiyami A
Ballum	Ballum	Mus 127
Bataviae	Bataviae	Swart
**Canicola**	**Canicola**	**Hond Utrecht IV**
**Grippotyphosa**	**Grippotyphosa**	**Moskva V**
**Icterohaemorrhagiae**	**Icterohaemorrhagiae**	**RGA**
Javanica	Javanica	Veldrat Batavia 46
Pomona	Pomona	Pomona
Pyrogenes	Pyrogenes	Salinem
Sejroe	Hardjo	Hardjoprajitno
Tarassovi	Tarassovi	Perepelitsin

The active components of the 4-serovar vaccine Nobivac^®^ L4 (MSD Animal Health, Boxmeer, The Netherlands) belong to the bolded serogroups.

**Table 3:** Characteristics of POC ^1^ tests for diagnosing canine leptospirosis according to manufacturers’ instructions.

POC Test	Manufacturer	Testing Principle	Detected Antibodies	Detected Serovars	Sample Material	Sample Volume [µL]
WITNESS^® 2^	Zoetis Delpharm Biotech, Lyon, France	Immunochromatographic test	IgM ^8^	Canicola Icterohaemorrhagiae Grippotyphosa Pomona	Serum Plasma Whole Blood	5
FASTest^® 3^	MegaCor Diagnostik, Hörbranz, Austria	Immunochromatographic test	IgM	n. a. ^10^	Serum Plasma Whole Blood	10
Test-it^TM 4^	Life Assay Diagnostics, Cape Town, South Africa	Immunochromatographic test	IgM	Icterohaemorrhagiae	Serum Whole Blood	5
ImmunoComb^® 5^	Biogal Galed Laboratories, Kibbutz Galed, Israel	Modified ELISA ^7^	IgG ^9^ (semi-quantitative)	Canicola Icterohaemorrhagiae Grippotyphosa Pomona	Serum Plasma Whole Blood	5–10
SNAP^® 6^	IDEXX Europe B.V., Hoofddorp, The Netherlands	ELISA	IgM, IgG	Canicola Icterohaemorrhagiae Grippotyphosa Pomona	Serum	10

^1^ POC test, point-of-care test; ^2^ WITNESS^®^, WITNESS^®^ Lepto (Zoetis); ^3^ FASTest^®^, FASTest^®^ LEPTOSPIRA IgM (MegaCor); ^4^ Test-it^TM^, Test-it^TM^ Leptospirosis (Life Assay); ^5^ ImmunoComb^®^, ImmunoComb^®^ Canine *Leptospira* Test Kit (Biogal); ^6^ SNAP^®^, SNAP^®^ Lepto (IDEXX); ^7^ ELISA, enzyme-linked immunosorbent assay; ^8^ IgM, immunoglobulin M; ^9^ IgG, immunoglobulin G; ^10^ n. a., not available.

**Table 4:** (**a**) Number of dogs (of a total of 97 dogs) with detected *Leptospira* spp. antibodies against vaccine and non-vaccine serovars by MAT ^1^ after revaccination. Only maximum antibody titres per dog and time point are included. (**b**) Median titres per serogroup and sampling time point of 97 dogs with detected *Leptospira* spp. antibodies against vaccine and non-vaccine serovars by MAT after revaccination. Corresponding frequency distributions per titre class are shown in Table 4a.

(**a**)						
**Antibody Titre**	**Week**					
	**0**	**2**	**4**	**12**	**26**	**52**
0	32	0	0	0	5	25
10	46	3	4	29	46	52
20	14	36	36	43	38	19
40	4	47	51	24	8	1
80	1	7	2	1	0	0
160	0	4	3	0	0	0
320	0	0	1	0	0	0
(**b**)						
**Serogroup**	**Median Antibody Titres in Week**					
	**0**	**2**	**4**	**12**	**26**	**52**
**Australis**	0	20	20	10	10	0
Autumnalis	0	10	10	0	0	0
Ballum	0	0	0	0	0	0
Bataviae	0	0	0	0	0	0
**Canicola**	0	20	20	10	10	0
**Grippotyphosa**	0	10	20	10	10	0
**Icterohaemorrhagiae**	0	10	10	10	10	0
Javanica	0	0	0	0	0	0
Pomona	0	0	0	0	0	0
Pyrogenes	0	0	0	0	0	0
Sejroe	0	0	0	0	0	0
Tarassovi	0	0	0	0	0	0

^1^ MAT, microscopic agglutination test. The active components of the 4-serovar vaccine Nobivac^®^ L4 (MSD Animal Health, Boxmeer, The Netherlands) belong to the bolded serogroups.

**Table 5:** Characteristics of POC ^1^ tests for diagnosing canine leptospirosis.

POC Test	Storage	Sample Material	Approximate Duration [Min]	Positive Result
WITNESS^® 2^	Room temperature	Serum Plasma Whole blood	2 (procedure) +10 (drying)	Control and test line visible
FASTest^® 3^	Room temperature	Serum Plasma Whole blood	2 (procedure) +15 (drying)	Control and test line visible
Test-it^TM 4^	Room temperature	Serum Whole blood	2 (procedure) +10 (drying)	Control and test line visible
ImmunoComb^® 5^	2–8 °C	Serum Plasma Whole blood	60–120 (thawing) +5 (procedure) +28 (drying)	Reference and test spot visible
SNAP^® 6^	2–8 °C	Serum	30 (thawing) +3 (procedure) +10 (drying)	Control and sample spot visible

^1^ POC test, point-of-care test; ^2^ WITNESS^®^, WITNESS^®^ Lepto (Zoetis); ^3^ FASTest^®^, FASTest^®^ LEPTOSPIRA IgM (MegaCor); ^4^ Test-it^TM^, Test-it^TM^ Leptospirosis (Life Assay); ^5^ ImmunoComb^®^, ImmunoComb^®^ Canine *Leptospira* Test Kit (Biogal); ^6^ SNAP^®^, SNAP^®^ Lepto (IDEXX).

**Table 6:** Number of positive dogs in *Leptospira* spp. antibody POC ^1^ tests and the reference method MAT ^2^ with a cut-off titre ≥ 80 after the revaccination of 97 dogs.

Test	Week					
	0	2	4	12	26	52
MAT	1	11	6	1	0	0
WITNESS^® 3^ (IgM ^4^)	1	18	10	1	2	1
FASTest^® 5^ (IgM)	1	13	7	1	1	1
Test-it^TM 6^ (IgM)	0	1	1	0	0	0
ImmunoComb^® 7^ (IgG ^8^)	32	81	78	66	49	42
SNAP^® 9^ (IgM, IgG)	13	43	34	21	13	12

^1^ POC test, point-of-care test; ^2^ microscopic agglutination test, reference titre regarded as positive; ^3^ WITNESS^®^, WITNESS^®^ Lepto (Zoetis); ^4^ IgM, immunoglobulin M; ^5^ FASTest^®^, FASTest^®^ LEPTOSPIRA IgM (MegaCor); ^6^ Test-it^TM^, Test-it^TM^ Leptospirosis (Life Assay); ^7^ ImmunoComb^®^, ImmunoComb^®^ Canine *Leptospira* Test Kit (Biogal); ^8^ IgG, immunoglobulin G; ^9^ SNAP^®^, SNAP^®^ Lepto (IDEXX).

**Table 7:** Comparison of *Leptospira* spp. antibody-detecting POC ^1^ tests (IgM ^2^ or IgG ^3^ exclusive or IgM/IgG combined) after the revaccination of 97 dogs using a mixed-effects logistic regression analysis. The *p*-values show the differences between the POC tests at different time points.

POC Test	Mixed-Effects Logistic Regression Analysis																	
	Week 0			Week 2			Week 4			Week 12			Week 26			Week 52		
	Odds Ratio	95% CI ^4^	*p*-Value	Odds Ratio	95% CI	*p*-Value	Odds Ratio	95% CI	*p*-Value	Odds Ratio	95% CI	*p*-Value	Odds Ratio	95% CI	*p*-Value	Odds Ratio	95% CI	*p*-Value
WITNESS^® 5^ (IgM) FASTest^® 6^ (IgM)	1.03	0.17–6.20	1	0.65	0.23–1.90	0.806	0.67	0.18–2.50	0.923	1.27	0.07–22	0.999	0.67	0.06–8.20	0.993	1.12	0.06–20.40	1
WITNESS^®^ (IgM) Test-it^TM 7^ (IgM)	0.10	0.01–2.90	0.339	0.05	0.01–0.60	**0.011**	0.09	0.01–1.30	0.105	0.08	0.01–34.90	0.790	0.04	0.01–16.50	0.600	0.07	0.01–36.70	0.782
FASTest^®^ (IgM) Test-it^TM^ (IgM)	10.30	0.52–204.40	0.205	13.30	1.04–169.90	**0.045**	7.82	0.51–120.30	0.241	15.80	0.06–4143.40	0.657	15.60	0.06–4243.90	0.668	15.10	0.05–4474.90	0.690
WITNESS^®^ (IgM) ImmunoComb^® 8^ (IgG)	37	6.08–225.70	**0.001**	22.40	8.07–62.40	**0.001**	35.90	11.53–11.90	**0.001**	190	15.68–2303	**0.001**	51	7.09–367	**0.001**	71.10	5.72–884.70	**0.001**
FASTest^®^ (IgM) ImmunoComb^®^ (IgG)	0.03	0.01–0.10	**0.001**	0.03	0.01–0.10	**0.001**	0.02	0.01–0.10	**0.001**	0.01	0.01–0.00	**0.001**	0.01	0.01–0.10	**0.001**	0.02	0.01–0.10	**0.001**
Test-it^TM^ (IgM) ImmunoComb^®^ (IgG)	373	16.37–8498.70	**0.001**	457	35.13–5932.40	**0.001**	420	28.53–6193.30	**0.001**	2360	8.20–680,335	**0.002**	1190	3.96–355,077.40	**0.006**	964	2.92–318,505.10	**0.011**
WITNESS^®^ (IgM) SNAP^® 9^ (IgM, IgG)	10.90	1.70–70.50	**0.004**	3.55	1.45–8.70	**0.001**	4.78	1.64–14	**0.001**	24.80	2.01–306.10	**0.005**	7.78	1–60.80	0.051	13.20	0.99–176.30	0.052
FASTest^®^ (IgM) SNAP^®^ (IgM, IgG)	0.09	0.02–0.40	**0.001**	0.18	0.07–0.50	**0.001**	0.14	0.04–0.40	**0.001**	0.05	0.01–0.40	**0.001**	0.09	0.01–0.60	**0.006**	0.09	0.01–0.70	**0.009**
Test-it^TM^ (IgM) SNAP^®^ (IgM, IgG)	110	4.71–2575.10	**0.001**	72.30	5.83–896.90	**0.001**	56	3.90–803.30	**0.001**	308	1.06–89,349	**0.046**	181	0.59–55,552.60	0.096	179	0.53–60,752.20	0.108
ImmunoComb^®^ (IgG) SNAP^®^ (IgM, IgG)	3.39	1.30–8.90	**0.005**	6.31	2.51–15.90	**0.001**	7.51	3.06–18.40	**0.001**	7.67	3.14–18.70	**0.001**	6.56	2.47–17.40	**0.001**	5.39	1.98–14.60	**0.001**

^1^ POC test, point-of-care test; ^2^ IgM, immunoglobulin M; ^3^ IgG, immunoglobulin G; ^4^ CI, confidence interval; ^5^ WITNESS^®^, WITNESS^®^ Lepto (Zoetis); ^6^ FASTest^®^, FASTest^®^ LEPTOSPIRA IgM (MegaCor); ^7^ Test-it^TM^, Test-it^TM^ Leptospirosis (Life Assay); ^8^ ImmunoComb^®^, ImmunoComb^®^ Canine *Leptospira* Test Kit (Biogal); ^9^ SNAP^®^, SNAP^®^ Lepto (IDEXX); bold values indicate statistically significant differences.

**Table 8:** Sensitivity and specificity for the POC ^1^ tests evaluated, calculated against MAT ^2^ cut-off titre ≥ 80 using all serovars analyzed by MAT at all study time points.

POC Test	Detected Antibodies *	Detected Serovars *	Positive Results	Negative Results	Prevalence	Sensitivity [%]	Specificity [%]	PPV ^3^ [%]	NPV ^4^ [%]	Accuracy [%]	Positive Likelihood Ratio	Negative Likelihood Ratio
WITNESS^® 5^	IgM ^10^	Canicola	33/582	549/582	5.67	100	97.50	70.63	97	91.75	1.91	0.95
		Icterohaemorrhagiae										
		Grippotyphosa										
		Pomona										
FASTest^® 6^		n. a. ^12^	24/582	558/582	4.12	100	99.10	82.68	97	93.30	2.69	0.93
Test-it^TM 7^		Icterohaemorrhagiae	2/582	580/582	0.34	100	99.80	63.04	97	96.39	0	97
ImmunoComb^® 8^	IgG ^11^	Canicola	348/582	234/582	59.79	100	41.60	71.80	100	43.13	1.62	0.13
SNAP^® 9^	IgM, IgG	Icterohaemorrhagiae	136/582	446/582	23.37	100	79.20	59.45	98	77.15	2.61	0.54
		Grippotyphosa										
		Pomona										

^1^ POC test, point-of-care test; ^2^ MAT, microscopic agglutination test; ^3^ PPV, positive predictive value; ^4^ NPV, negative predictive value ^5^ WITNESS^®^, WITNESS^®^ Lepto (Zoetis); ^6^ FASTest^®^, FASTest^®^ LEPTOSPIRA IgM (MegaCor); ^7^ Test-it^TM^, Test-it^TM^ Leptospirosis (Life Assay); ^8^ ImmunoComb^®^, ImmunoComb^®^ Canine *Leptospira* Test Kit (Biogal); ^9^ SNAP^®^, SNAP^®^ Lepto (IDEXX); ^10^ IgM, immunoglobulin M; ^11^ IgG, immunoglobulin G; ^12^ n. a., not available. * according to manufacturer’s instructions.

## Data Availability

The raw data supporting the conclusions of this article will be made available by the authors on request.
